# Early weight-bearing after acetabular fractures in the older patient: a systematic review

**DOI:** 10.1530/EOR-2024-0191

**Published:** 2025-09-04

**Authors:** Marte I Lommerse, Anna H M Mennen, Frank W Bloemers, Hanna C Willems, Daphne van Embden

**Affiliations:** ^1^ Department of Trauma Surgery, Amsterdam UMC, Amsterdam, The Netherlands; ^2^ Amsterdam Bone Center, Amsterdam Musculoskeletal Sciences, Amsterdam UMC research institute, Amsterdam, The Netherlands; ^3^ Department of Internal Medicine and Geriatrics, Amsterdam UMC, Amsterdam, The Netherlands

**Keywords:** early weight-bearing, acetabular fracture, older patients, rehabilitation

## Abstract

**Purpose:**

- While the incidence of acetabular fractures keeps rising among our older patient population, age-specific rehabilitation guidelines are lacking. Post-surgery weight-bearing is often restricted for 8–12 weeks to avoid secondary fixation failure. However, non- or restricted weight-bearing commonly results in atrophy, and older patients are at additional risk of long-term mobility and functionality loss. Therefore, if the risk of secondary fracture failure proves to be lower than currently believed, early or permissive weight-bearing might actually be the preferred treatment choice to advance fracture healing and decrease recovery time. This study aims to review the current literature on early weight-bearing outcomes of acetabular fractures in older adults.

**Methods:**

- A systematic search of two databases was conducted following PRISMA guidelines. Eligible studies reported on early weight-bearing outcomes of older patients after surgical management. Data were showed in tables alongside a narrative synthesis. Critical appraisal and risk-of-bias tools assessed the study quality.

**Results:**

- Six studies were included with a retrospective or observational design, with a total of 147 patients averaging 64 years old. The majority of studies discussed early weight-bearing protocols after percutaneous fixation (PF), with heterogeneity in terminology across protocols.

**Conclusions:**

- Albeit limited and low in evidence, the included studies suggest that early weight-bearing might be a possible alternative for non- or minimally displaced fractures and displaced fractures after PF and open reduction internal fixation, respectively. When risk assessments and functional outcomes are appropriately investigated, early weight-bearing may help patients, especially older adults, avoid suffering and prolonged rehabilitation periods.

## Introduction

When discussing the care and treatment of acetabular fractures, advanced age has been described as a major predictor of inferior outcomes (1). This is reflected by a rate of secondary hip replacement of 23% in those over 60 years, a 1-year mortality rate of 11% (2), and a 25% increase in this rate in the case of concomitant injuries (3). Simultaneously, due to a growing older patient population with an increasingly active lifestyle, incidence numbers of acetabular fractures are rising exceedingly fast (2, 4, 5). Unfortunately, treatment and rehabilitation guidelines are not developing at the same pace. The current standard protocol of 8–12 weeks of restricted weight-bearing after initial treatment (6) does not accommodate the unique challenges that older patients face during recovery. A different set of physiological processes and comorbidities, along with distinct fracture patterns and associated injuries, all contribute to the complexity of fracture treatment and rehabilitation in the older patient population.

When treating more porous bones and complex fracture patterns, as is the case in older patients, fear of implant failure and fracture displacement undoubtedly remains a valid argument (5, 7). However, the recent literature actually reports that the risk-averse approach to weight-bearing protocols could negatively impact the return to pre-existing functionality for older patients due to cumulating comorbidities and decreasing resilience that results from long periods of being bed- or chairbound (8, 9). Starting to bear weight sooner might actually evoke micro-movements between fracture fragments that could stimulate fracture healing during the early stages of rehabilitation (10). Moreover, numerous studies illustrate a lack of compliance in restricted weight-bearing protocols. Even with real-time feedback devices (11), many patients exceed the prescribed amount of weight-bearing often anyway. Interestingly, these studies do not record increased complications due to fracture overloading. Therefore, the aim of this study is to review the current literature on early weight-bearing in acetabular fractures in older adults.

## Methods

A systematic search was carried out according to Preferred Reporting Items for Systematic Reviews and Meta-Analyses (PRISMA) guidelines (12). Ethics approval was not needed because all analyses were done with data from previously published studies.

### Search strategy and study selection

The electronic databases of Pubmed/MEDLINE and Embase were searched on August 7th, 2024. Various combinations of medical subject headings and title/abstract terms related to ‘acetabulum fracture’ and ‘weightbearing’ were used during the systematic search using database-appropriate search phrases (see Supplemental file I (see section on Supplementary Materials given at the end of the article)). To collect further relevant citations, references and review articles were hand-screened. Prospective, retrospective, and comparative studies (RCT’s), as well as observational and case report studies, were included. Initially, we planned to include only studies with patients aged 50 and older; however, this resulted in too few articles to analyze, jeopardizing the robustness of this review. Therefore, studies that included some younger patients were also included. The median age of patients included in these articles was calculated when possible to assess whether the age distribution was skewed toward older patients. Articles focusing on included patients treated with total hip arthroplasty (THA) were excluded, as well as studies of patients with pathological fractures. Other exclusion criteria were failure to report evaluation outcomes, cadaveric or animal studies, biomechanical testing, review studies, or articles only discussing surgical techniques. No randomized studies were available on this topic. After eliminating duplications, a total of 1,182 studies were screened independently by two reviewers (ML and AM) based on title and abstract using Rayyan.AI software. Afterward, full texts of all potentially eligible articles were acquired and reviewed by both. Disagreements regarding inclusion were resolved by a third author (DE).

### Data extraction and synthesis

Data extraction was performed by one reviewer (ML) using Microsoft Excel. Extracted data included first author, publication year, study population and period, design, several patient demographics, fracture classification according to Judet & Letournel (13), treatment (surgical/non-surgical, surgery type), weight-bearing protocol specifications (full/toe-touch/restricted, immediate/early/late), follow-up time, functional (walking distance, VAS scores, Modified Merle d’Aubigne (mMA) score), and radiological outcomes (anatomical/near-perfect/poor fracture reduction), complications, and information to assess the risk of bias in each study. When available, continuous variables were showed as means with standard deviations. Categorical variables were showed as percentages. Variables affected by missing data were identified and clearly reported in the synthesis. A narrative synthesis of the results is showed below. The quality of the included studies was assessed using the GRADE tool (14) and the ROBINS-I risk of bias tool (15).

## Results

### Study selection and characteristics

**Figure 1:**
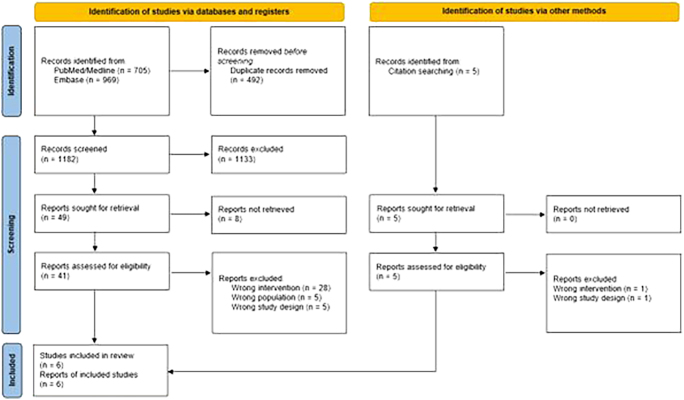
The PRISMA flow-diagram for the literature search of this systematic review (12).

**Figure 2:**
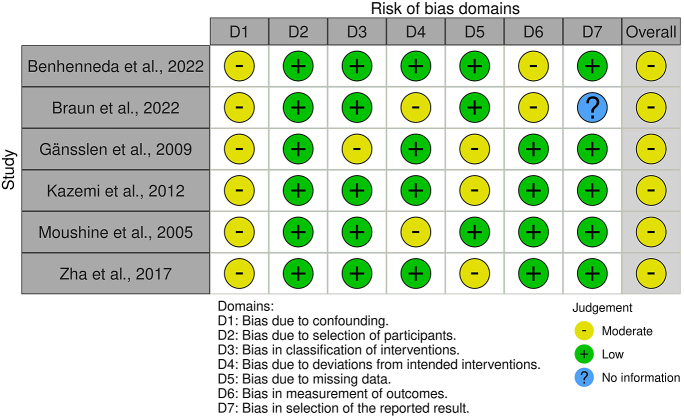
Risk of bias in included studies (16, 17, 18, 19, 20, 21) as assessed using ROBINS-I risk of bias tool (15).

In total, 1,182 abstracts were screened. A total of six studies (147 patients) were included in the systematic review after screening. Article inclusion details are showed in the PRISMA flowchart (Fig. 1). In the final analysis, only observational retrospective cohort studies were included. The quality of evidence was graded either low (*n* = 4) or very low (*n* = 2). Risk of bias was assessed as moderate for all studies. The majority of studies specifically focused on early weight-bearing after percutaneous fixation (PF), while the remainder of studies included both PF and open reduction and internal fixation (ORIF) as primary fracture management. Quality and risk-of-bias assessments were performed on all studies and are showed in Table 1 and Fig. 2. Further study characteristics have been summarized in Table 2.

**Table 1:** GRADE evidence profile of included studies.

Studies	Design	Risk of bias	Inconsistency	Indirectness	Imprecision	Publication bias	Overall LOE
Benhenneda *et al.* (16)	OB-RS	Moderate	Low	Low	Low	None	Low ⊕⊕◯◯
Braun *et al.* (17)	OB-PS	Moderate	Unclear*	Low	Moderate^§^	None	Very low ⊕◯◯◯
Gänsslen & Krettek (18)	OB-RS	Moderate	Unclear*	Moderate^‡^	Low	None	Low ⊕⊕◯◯
Kazemi *et al.* (19)	OB-RS	Moderate	Moderate^†^	Low	Moderate^§^	None	Very low ⊕◯◯◯
Mouhsine *et al.* (20)	OB-RS	Moderate	Low	Moderate^‡^	Low	None	Low ⊕⊕◯◯
Zha *et al.* (21)	OB-RS	Moderate	Low	Moderate^‡^	Low	None	Low ⊕⊕◯◯

* Patient characteristics not clearly stated.

^†^ Wide variety between patients.

^‡^ Indirect outcome measure.

§ Wide confidence interval for outcome measure.

OB-RS, observational retrospective; OB-PS, observational prospective; LOE, level of evidence.

**Table 2:** Details of studies and patient demographics included in the systematic review.

Study characteristics	Benhenneda *et al.* (16)	Braun *et al.* (17)	Gänsslen & Krettek (18)	Kazemi *et al.* (19)	Mouhsine *et al.* (20)	Zha *et al.* (21)	Total
Study objective	Pelvic/acetabular #, PF, IFWB	Acetabular #, post-op, EWB compliance	Acetabular #, ORIF, PWB	Acetabular #, PF, IFWB	Acetabular #, PF	Acetabular #, age effect on ORIF outcomes	
Total, *n*	27	10	27	28	21	53	166
Acetabular fractures, *n*	8	10	27	28	21	53	147
Males, *n*	NA	NA	19	21	12	44	96 (74.4%)
Mean age (range)	75.5 (65–97)	62.2 (25–84)	43.5 (17–76)	49 (18–83)	81 (67–90)	72.8 (60–90)	
Pts with comorbidities, *n*	8	NA	NA	12	NA	52	72 (80.9%)
Pts functioning independently pre-injury, *n*	NA	NA	NA	27	21	NA	48 (98%)
Mean LOS, days (range)	5 (3–12)	NA	NA	7 (2–38)	9 (7–12)		
Mean FU, months (range)	1.5	1.5	24	39 (1–74)	42	52.5 (18–110)	
Pts lost to FU, *n*	0	0	10	6	1	7	24

Pts, patients; PF, percutaneous fixation; IFWB, immediate full weight-bearing; EWB, early weight-bearing; ORIF, open reduction internal fixation; PWB, permissive weight-bearing; na, not available; LOS, length of hospital stay.

### Patient and fracture characteristics

The study participants ranged from eight patients to 53. The weighted mean age of all patients was 63.5 years (range: 18–97). When only sampling the studies with an exclusively older population (16, 20, 21), the 82 patients had a weighted mean age of 75.2 years (range: 60–97). Four out of six studies (18, 19, 20, 21) showed a male-female relationship (129 patients), with 33 (25.6%) females and 96 (74.4%) males. In order to estimate a patient’s frailty status, comorbidities and pre-injury level of activity were evaluated. Three studies (16, 19, 21) reported comorbidities or ASA scores (89 patients), of whom 72 patients (80.9%) had comorbidities or an ASA score of 2 or higher. Two studies (19, 20) included their patients’ pre-injury activity level (48 patients), and all but one patient were independently walking pre-injury.

In Table 3, fracture classification according to the Letournel classification is showed. The most common fracture patterns were both-column fractures (16.9%), anterior column or wall fractures (14.0%), and anterior column and posterior hemi-transverse fractures (12.2%). Injury mechanism was showed in four studies (18, 19, 20, 21). Seventy-one patients sustained a high-energy trauma (HET, 55%), versus 54 patients who sustained a low-energy trauma (LET, 41.9%). Four patients (3.1%) could not be categorized. Fifty-two patients (40.3%) sustained associated injuries. Fifty-seven patients (38.8%) were treated with PF, versus 90 patients (61.2%) with ORIF. The overall level of fracture reduction, meaning how well the fracture had been surgically reduced, was postoperatively assessed using either three-plane radiography films (20, 21) or pelvic CT scans (18, 19). In the four articles in which this parameter was included, reduction was anatomic in 69%, imperfect in 21.7%, and poor in 6.2%. The mean length of stay was reported in three studies (16, 19, 20) and was 7 days (IQR: 6; 7; 8). Further specifics regarding the injury mechanism and surgery outcomes are showed in Table 4.

**Table 3:** Distribution of fractures in the included studies according to Letournel classification.

Fracture type	Values, *n* (%)
Posterior wall	7 (4.8)
Anterior wall	4 (2.7)
Posterior column	3 (2)
Anterior column	19 (12.9)
Transverse	12 (8.2)
Posterior wall and posterior column	2 (1.4)
Transverse and posterior wall	7 (4.8)
T-shaped	15 (10.2)
Anterior with posterior hemi transverse	26 (17.7)
Both columns	52 (35.4)
Total	147

**Table 4:** Injury characteristics, surgical details, and outcomes of the included studies.

Characteristics	Values, *n* (%)
Injury mechanism	
LET	54 (36.7)
HET	71 (48.3)
Not specified	4 (2.72)
NA	18
Associated injuries	
Orthopedic	29 (19.7)
Head	6 (4.08)
Thorax	8 (5.44)
Abdomen	5 (3.40)
Neurological	3 (2.04)
NA	18
ORIF	90 (61.2)
PF	57 (38.8)
Radiological outcome*	
Anatomic	89 (60.5)
Imperfect (<2 mm)	28 (19.1)
Poor	8 (5.44)
NA	22

* Level of reduction.

LET, low energy trauma; HET, high energy trauma; ORIF, open reduction internal fixation; PF, percutaneous fixation; na, not available.

### Clinical outcomes of early weight-bearing

All included studies specified their early weight-bearing protocol, the details of which are summarized in Table 5. Noticeably, most studies used different terms in their early weight-bearing protocols. Furthermore, the terms that were used (e.g., touch-down, partial, permissive) were often not explicitly defined. Patients who underwent PF were among the first to start with full weight-bearing. Two studies (16, 19) had all their patients (36 patients, 24.5%) start weight-bearing immediately with 100% weight. The other studies (111 patients, 75.5%) had their patients start partial weight-bearing within 2 days after surgery. This resulted in a mean time to full weight-bearing of 7.3 weeks (SD: 4.16 weeks) for the 111 patients with restrictions. Functional recovery was reported differently among the articles. Most (18, 19, 20, 21) referred to the mMA score (129 patients, 87.8%), while one study (8 patients, 5.44%) described functional recovery in terms of walking distance (16), and another in terms of weight-bearing force (17). Of the 129 patients in which mMA score was used as the outcome measurement, the mean at the end of follow-up was 16.7 points (ICR: 16.4; 16.9; 17.2). Only one of the included studies showed secondary fracture displacement (2 patients, 1.36%). Zha *et al.* (21) reported two cases of displacement, in which conversion to THA was performed at 30 and 35 months after the initial injuries, respectively. The first patient had sustained a femoral head injury and subsequent osteonecrosis of the head, while the second had poor reduction and subsequent osteoarthritis. Zha *et al.* furthermore reported two cases of reduction loss where conversion to THR was not deemed necessary, resulting in 2.72% secondary fracture progression in all included studies. Other complications showed by the different studies included arthrosis, heterotopic ossification, one case of nonunion, wound infection, and deep venous thrombosis (26 patients, 17.7%). Follow-up ranged from 6 to 210 weeks. A total of eight patients (5.44%) were reported to have died during the follow-up period of the included studies. In studies where the reason for death was made available, none of the deaths were considered (in-)direct results of early weight-bearing.

**Table 5:** Details of weight-bearing protocol, primary outcome, and complications per study.

	Benhenneda *et al.* (16)	Braun *et al.* (17)	Gänsslen & Krettek (18)	Kazemi *et al.* (19)	Mouhsine *et al.* (20)	Zha *et al.* (21)	Total
Fracture type	Pelvis & acetabulum	Acetabulum	Acetabulum	Acetabulum	Acetabulum	Acetabulum	
Acetabular fractures, *n*	8	10	27	28	21	53	147
Fixation construct	PF	ORIF	ORIF	PF	PF	ORIF	
Weight-bearing protocol	IFWB	PWB (20 kg)	PWB (15 kg)	IFWB	TTWB	TTWB (20 kg)	
Duration		Post-op until 6 weeks	48 h post-op until 8–12 weeks		24 h post-op until 4 weeks	48 h post-op until 12 weeks	
Mean time to FWB, weeks	0	6	NA	0	4	12	
Conversion to THA	0	0	0	0	0	2	2 (1.36%)
Other complications	0	NA		0	0		28 (16.8%)
Arthrosis			3				
Heterotopic ossification			2			12	
Nerve palsy						2	
DVT						2	
Infection						1	
Femoral head necrosis						2	
Incisional hernia						1	
Mortality	3	0	0	1	2	2*	8 (5.44%)

PF, percutaneous fixation; ORIF, open reduction internal fixation; IFWB, immediate full weight-bearing; PWB, partial weight-bearing; PT, physiotherapy; TTWB, toe-touch weight-bearing; WBAT, weight-bearing as tolerated; NA, not available.

* Patients were excluded from the study as death due to natural causes occurred within 12 months of follow-up.

## Discussion

Poor reduction and secondary fracture displacement after acetabular fractures are associated with inferior patient outcomes, increased risk of development of posttraumatic arthritis, and conversion to THA (22). A second surgery is associated with an increased risk of peri- and post-surgical complications due to a different anatomy post-surgery and a relatively short recuperation period for patients (23). For older patients, the impact a second surgery has is even greater due to an overall lower resilience and less strength to recover (24). Taken together, secondary fracture displacement would be a major complication of early weight-bearing, which could explain why only a limited number of studies that permit early weight-bearing after acetabular fractures in the elderly have been carried out.

From a biomechanical perspective, the literature suggests that early weight-bearing might be advantageous in treating acetabular fractures. Several articles reported increased pressures on the acetabulum during non-restricted sit-to-stand movements when compared to normal ambulation (25, 26). According to Yoshida *et al.* (26), peak pressures in these sit-to-stand movements are directed toward the posterior wall of the acetabulum, which results in an increase of 2.8 times more than during ambulation. Another study showed a fourfold increase in required energy for ambulation under weight-bearing restrictions versus during full weight-bearing (27). In this article, 11 healthy young patients were subjected to either full, toe-touch, or non-weight-bearing, with results demonstrating an increased energy cost in toe-touch and non-weight-bearing compared to normal walking. These biomechanical studies point toward the suggestion that early non-restricted weight-bearing might be favorable in rehabilitation of acetabular fractures. Berk *et al.* conducted a biomechanical study comparing ORIF and screwable cups for THA (28). Despite limitations in sample size and artificial bone use, their findings suggest potential safety for full weight-bearing after ORIF.

When put in a clinical context, it is widely accepted that stimulating movement is an essential aspect of recovery from any illness. Complications such as bedsores and muscle atrophy are very common in patients with prolonged hospitalization and contribute to an extended recovery period (8). In older patients, this could, in turn, result in higher fall risk and even more fractures or other injuries, with considerably lower return-to-home rates and a higher demand for nursing homes and homecare nurses (9). Finally, immobility in the older patient inevitably results in higher mortality rates (29). Unfortunately, clinical evidence on early weight-bearing outcomes remains limited. This study concentrates on older patients; however, the existing literature regarding early weight-bearing following acetabular fractures is scarce and typically includes a broader patient demographic. Of the three articles also including younger patients, only one provided additional data from which a median age could be calculated. While in Braun *et al.* (17) the median age was indeed higher than the average age (64.5 versus 62.2 years), the other two articles (18, 19) did not provide any information regarding age distribution. Excluding these studies from our analysis would have significantly reduced the data available for review, limiting the robustness of our conclusions. However, we acknowledge that outcomes may vary depending on the bone quality, healing potential, and functional needs of younger and older patients.

A review by Murena *et al.* (10) found evidence supporting early weight-bearing after PF. Multiple authors allowed early weight-bearing of non- or minimally displaced (<2 mm) anterior column and anterior column posterior hemitransverse fractures after PF and demonstrated no secondary displacement. For example, Bozzio *et al.* (30), in their review, reported good clinical outcomes and low secondary displacement risk after weight-bearing as tolerated with crutches or a walker. Furthermore, Caviglia *et al.* (31) directed patients to begin 50% weight-bearing 2 days after surgery. However, the weight-bearing protocols in these studies were not specifically aimed at older patients, as they included HET and LET patients of all ages. It should furthermore be noted that the positive results could better be attributed to the stability of these specific fractures due to minimal or even no displacement before surgery. This could, for example, be the case in two studies included in this review (19, 20), which both only performed PF on minimally displaced fractures. Nonetheless, a number of studies reported positive results in displaced fractures (>2 mm), both treated percutaneously and with posterior or anterior ORIF surgery. In a systematic review comparing 152 cases of early (<12 weeks) versus 302 cases of late weight-bearing (>12 weeks), no differences regarding functional scores or complication rates were found (32). The total cohort consisted of patients who had sustained surgically managed posterior wall fractures. Both Zha *et al.* (21) and Gänsslen & Krettek (18) included patients with comparable fractures and came to similar functional outcomes. In all included studies, only two patients underwent THA secondary to their ORIF surgery, and two others suffered secondary fracture displacement without conversion surgery. While data from the included studies were too heterogeneous to be combined in a meta-analysis, the literature suggests that early weight-bearing might be a good alternative in rehabilitation, even in displaced fractures followed by ORIF surgery.

There are several aspects that make it difficult to integrate a structured and methodized concept of early weight-bearing into clinical practice. Apart from the evidence hiatus on safety and functional outcomes in the literature, the different terms used when discussing weight-bearing (e.g., partial, permissive, toe-touch) are not clearly defined. Recently, a terminology consensus meeting was held for all major stakeholders –including patients– in the field of rehabilitation of weight-bearing fractures in the United Kingdom (33). Not only were the current descriptors defined as unclear by all participants in the study, but there was consensus on the use of ‘non-weight-bearing’/‘limited weight-bearing’/‘unrestricted weight-bearing’ as the most fitting terminology for weight-bearing instructions. As the authors of the British Weight-bearing Consensus Working Group also note, uniform language could improve research fidelity and provide more clarity in clinical protocols for both patients and healthcare providers. In clinical practice, the weight-bearing protocol is still primarily based on a surgeon’s experience, certain patient characteristics, and a ‘gut feeling’ relating to the frailty of the patient and the fracture itself. Furthermore, low patient compliance in weight-bearing protocols is thought to be a significant issue, with evidence suggesting a lack of compliance in restricted and partial weight-bearing protocols. Oftentimes, patients are told to bear weight a certain amount of kilograms or are guided by pain levels, which seem difficult to adhere to (11). In a recent prospective multicenter cohort study (34), early weight-bearing protocols for the lower extremities were developed and applied to 106 patients of all ages. Although this cohort included patients with acetabular fractures, these were grouped together with pelvic fractures and were therefore not suitable for inclusion in this review. However, the study introduced a patient-centered early weight-bearing protocol that demonstrated positive outcomes in terms of quality of life and activities of daily living across all fracture types. Given the lack of specific data on acetabular fractures in older patients, a dedicated prospective study investigating early weight-bearing in this patient group would be of significant interest.

### Limitations

Several important limitations of this study have to be taken into account. All included studies were retrospective and were very heterogeneous on several fronts. First of all, multiple studies included patients from all ages, sample sizes were small, and no study included comparison groups. The inclusion of studies with broader age demographics provides the most important challenge for interpretation of this study’s outcomes. Moreover, some studies did not report characteristics such as gender, comorbidities, injury type, and associated injuries. In this regard, only one article included a supplementary table of detailed patient information. This missing data could possibly introduce reporting bias. Inadequate or missing details on outcomes per patient age group and fracture pattern limit the clinical precision with which our study results can be interpreted. Furthermore, different outcome measures were used to investigate radiological and functional outcomes. Some studies did not include any radiological or functional outcome measurements. This variability or absence in outcome parameters limits the reliability of the results. Finally, the use of terms such as restricted, permissive, as tolerated, toe-touch, and full weight-bearing were oftentimes not explicitly defined, possibly leaving room for different interpretations of these terms by included patients, authors, or readers. This possible variability in weight-bearing strategy could therefore influence functional outcomes in the included studies, as well as our interpretation of the results.

## Conclusion

The management of acetabular fractures in older adults presents a unique challenge for all medical professionals involved in their care. Treatment and rehabilitation strategies are, due to an absence of formal guidelines, varied and require individualization based on different patient and injury factors. There is limited evidence suggesting that early weight-bearing provides similar outcomes and might be a possible alternative for non- or minimally displaced fractures and displaced fractures after PF and ORIF, respectively. Furthermore, early weight-bearing could be advantageous for early recovery without increased risk of secondary fracture displacement, THA-conversion, and posttraumatic arthritis. While our outcomes are not clear-cut, the highlighted lacunae in current knowledge present new areas of interest and future possibilities that could improve the recovery process of acetabulum fractures in older adults.

## ICMJE Statement of Interest

The authors declare that there is no conflict of interest that could be perceived as prejudicing the impartiality of the work reported.

## Funding Statement

The study was funded by a grant from Zorg Onderzoek Nederland en Medische Wetenschappen (ZonMW) Foundation. ZonMW had no involvement in the research process.

## Author contribution statement

MI Lommerse contributed to conceptualization, data curation, writing the original draft and writing review and editing. AHM Mennen contributed to data curation and writing reviews and editing. D van Embden, HC Willems contributed to conceptualization and writing review and editing. FW Bloemers contributed to writing review and editing.
